# Quantifying the Disadvantage of Small Recipient Size on the Liver Transplantation Waitlist, a Longitudinal Analysis Within the Eurotransplant Region

**DOI:** 10.1097/TP.0000000000004804

**Published:** 2023-11-13

**Authors:** Dimitri Sneiders, Anne-Baue R. M. van Dijk, Sarwa Darwish-Murad, Marieke van Rosmalen, Nicole S. Erler, Jan N. M. IJzermans, Wojciech G. Polak, Hermien Hartog

**Affiliations:** 1 Department of Surgery, Division of HPB and Transplant Surgery, Erasmus MC Transplant Institute, Erasmus University Medical Center, Rotterdam, the Netherlands.; 2 Department of Gastroenterology and Hepatology, Erasmus MC Transplant Institute, Erasmus University Medical Center, Rotterdam, the Netherlands.; 3 Eurotransplant International Foundation, Leiden, the Netherlands.; 4 Department of Biostatistics, Erasmus University Medical Center, Rotterdam, the Netherlands.; 5 Department of Epidemiology, Erasmus University Medical Center, Rotterdam, the Netherlands.; 6 Department of HPB and Liver Transplantation, University Medical Center Groningen, Groningen, the Netherlands.

## Abstract

**Background.:**

Small adult patients with end-stage liver disease waitlisted for liver transplantation may face a shortage of size-matched liver grafts. This may result in longer waiting times, increased waitlist removal, and waitlist mortality. This study aims to assess access to transplantation in transplant candidates with below-average bodyweight throughout the Eurotransplant region.

**Methods.:**

Patients above 16 y of age listed for liver transplantation between 2010 and 2015 within the Eurotransplant region were eligible for inclusion. The effect of bodyweight on chances of receiving a liver graft was studied in a Cox model corrected for lab-Model for End-stage Liver Disease (MELD) score updates fitted as time-dependent variable, blood type, listing for malignant disease, and age. A natural spline with 3 degrees of freedom was used for bodyweight and lab-MELD score to correct for nonlinear effects.

**Results.:**

At the end of follow-up, the percentage of transplanted, delisted, and deceased waitlisted patients was 49.1%, 17.9%, and 24.3% for patients with a bodyweight <60 kg (n = 1267) versus 60.1%, 15.1%, and 18.6% for patients with a bodyweight ≥60 kg (n = 10 520). To reach comparable chances for transplantation, 60-kg and 50-kg transplant candidates are estimated to need, respectively, up to 2.8 and 4.0 more lab-MELD points than 80-kg transplant candidates.

**Conclusions.:**

Decreasing bodyweight was significantly associated with decreased chances to receive a liver graft. This resulted in substantially longer waiting times, higher delisting rates, and higher waitlist mortality for patients with a bodyweight <60 kg.

Small adult patients with end-stage liver disease waitlisted for liver transplantation may face a shortage of size-matched liver grafts.^1-5^ This may result in longer waiting times, increased waitlist removal, and increased waitlist mortality for this patient group. Additionally, this has been postulated as one of the reasons female transplant candidates have impaired access to liver transplantation compared with male candidates.^6-8^ Access to liver transplantation for small adults may therefore be lower as compared with average-sized patients.

In the Eurotransplant region, donor liver grafts are allocated based on recipient urgency with the use of the Model for End-stage Liver Disease (MELD) score. After allocation based on the MELD score, a large discrepancy (eg, weight difference >20%) of the donor exceeding the recipient size may result in rejection of the donor offer, due to concerns that a large liver graft may result in complex implantation and compression of blood supply or venous outflow, potentially resulting in graft loss.^9-12^

Previous studies with data from the United States and United Kingdom showed a disadvantage for low-weight candidates compared with average-weight individuals, which was most profound in adult-only transplantation centers.^1-4^ Splitting a liver for use in a pediatric and adult recipient or for 2 smaller adult recipients has been proposed previously and may reduce waitlist mortality on the pediatric waitlist, as well as improving access to liver transplantation for small adult recipients.^13,14^

Data on a potential disadvantage for small adult recipients within the Eurotransplant region have been lacking. Therefore, the primary objective of the current study is to assess access to liver transplantation in small adult liver transplant candidates throughout the Eurotransplant region as compared with normal-/large-sized candidates.

## MATERIALS AND METHODS

This study was approved by the institutional review board of the Erasmus MC University Medical Centre, Rotterdam. This study was conducted according to the STROBE guidelines.^15^

### Study Design

Adult patients (>16 y) listed for primary adult liver transplantation registered within the Eurotransplant region between 2010 and 2015 were eligible for inclusion. This period was chosen to ensure most patients were either transplanted or delisted, and few patients remained waitlisted at the end of follow-up. Patients listed for high-urgency liver transplantation and approved organ combination were excluded. Patients who received LDLT, suffered from polycystic liver disease, or who were classified as acute liver failure were also excluded. In patients with polycystic liver disease, the subdiaphragmatic space is usually substantially enlarged, rendering recipient and donor size matching less relevant. For descriptive statistics, patients with a bodyweight <60 kg were compared with patients with a bodyweight ≥60 kg. For the primary longitudinal survival analysis, no specific weight cutoff was chosen, and patient weight was fitted as a continuous variable.

### Outcomes

The primary outcome was defined as time to liver transplantation. Secondary outcomes included mortality on the waitlist, delisting from the waitlist, reasons for delisting, actual donor–recipient bodyweight matching, use of split transplantation, use of exception MELD points, national MELD score at delisting, and posttransplantation survival.

### Data Collection

All data analyzed (ie, age, sex, bodyweight, and laboratory values) were provided by Eurotransplant International Foundation, which covers the transnational organ allocation within Austria, Belgium, Croatia, Germany, Hungary, Luxemburg, the Netherlands, and Slovenia with a population of 135 million inhabitants. Data were extracted from the prospectively maintained Eurotransplant database. Apart from study outcomes, accessed data included recipient bodyweight and height. All lab-MELD score updates from the moment of listing up to the moment of delisting were extracted with the accompanying time interval. Other data included patient sex, age, blood type, indication for transplantation (alcohol-related liver disease, hepatitis C virus/hepatitis B virus-related liver cirrhosis, cryptogenic liver cirrhosis, cholestatic liver disease, malignancy, or other liver disease), and country of listing.

### Statistical Analysis

Statistical analysis was performed with R (version 4.0). Continuous variables were presented as median and interquartile range. Discrete variables were presented as absolute numbers and percentage. The overall proportion of missing data was low (maximum 3.9% for a single variable); therefore, a complete case analysis was performed, and no measures were taken to account for missing data in the analysis. The proportion of patients who received a transplant who were delisted or died until the end of follow-up were calculated. For illustrative purposes, time to transplantation, delisting, and mortality were depicted in stacked cumulative incidence plots according to the cause-specific hazards method. The primary outcome, time to liver transplantation, was studied in a cause-specific Cox model with lab-MELD updates fitted as time-dependent variable with a natural cubic spline with 3 degrees of freedom to account for nonlinear effects. To assess the effect of recipient weight on transplantation chance, corrected for lab-MELD score, recipient weight at initial listing was included in the model using a natural cubic spline with 3 degrees of freedom, as well as the interaction between the splines for weight and the lab-MELD score. The model was further corrected for blood type, age, and malignant disease as listing indication. The proportional hazard assumption was assessed with Schoenfeld residuals plots. Models were refitted with 4 and 5 (instead of 3) degrees of freedom for the splines to assess if this further improved the model concordance. Effect plots were used to visualize the estimated nonlinear effects, relative to a reference patient (weight 80 kg, blood type A, nonmalignant indication, aged 56). For illustrative purposes, the hazard ratio (chance for transplantation) for 50-kg, 55-kg, and 60-kg patients as compared with a 80-kg patient was estimated for varying lab-MELD scores based on the cause-specific Cox model. Additionally, lab-MELD scores required for theoretical 50-kg and 60-kg patients to attain similar transplant chances as 80-kg patients were calculated based on the log hazard estimate from the Cox model, and calculations were made for lab-MELD scores between 10 and 30 with incremental steps of 5 points. Posttransplant graft and patient survival was presented in Kaplan–Meier graphs for patients <60 kg versus patients ≥60 kg- and for patients <60 kg with >20% versus ≤20% weight difference to their donors and compared with log-rank tests. Graft survival was calculated as time to either graft loss or patient morality.

## RESULTS

### Characteristics of Waitlisted Patients

Characteristics of waitlisted patients are summarized in Table 1. In total, 1267 out of 11 787 (10.7%) patients with a bodyweight below 60 kg were listed. The majority of patients with a low bodyweight were female (78.0% female versus 25.2% male). Blood types were equally distributed in patients with a low and average bodyweight. Patients with a low bodyweight were more often listed for cholestatic liver disease (20.2% versus 10%) and less often for malignant liver disease (12.6% versus 24.1%). Patients with a low bodyweight were more often listed in a combined adult-pediatric transplant center (41.3% versus 36.8%). In the same period 484 out of 6972 (6.9%) donors had a bodyweight below 60 kg.

**TABLE 1.** Patient characteristics and characteristics of transplanted patients

	Weight ≥60 kg	Weight <60 kg
Patient characteristics		
n	10 520	1267
Age	56 (49–62)	53 (44–60)
Sex (female)	2654 (25.2)	988 (78.0)
Blood type		
O	3931 (37.4)	464 (36.6)
A	4550 (43.3)	563 (44.4)
AB	559 (5.3)	69 (5.4)
B	1485 (14.1)	171 (13.5)
Indication		
Alcoholic liver cirrhosis	3291 (31.3)	360 (28.4)
HCV/HBV-related liver disease	1487 (14.10)	157 (12.4)
Cryptogenic liver cirrhosis	720 (6.8)	89 (7.0)
Cholestatic liver disease	1053 (10.0)	256 (20.2)
Malignant liver disease	2535 (24.1)	160 (12.6)
Other	1434 (13.6)	245 (19.3)
Listing lab-MELD score	14 (10–20)	14 (9–19)
Listing country		
Germany	6491 (61.7)	803 (63.4)
Belgium	1302 (12.4)	149 (11.8)
Austria	856 (8.1)	89 (7.0)
Croatia	749 (7.1)	63 (5.0)
Netherlands	712 (6.8)	59 (4.7)
Hungary	295 (2.8)	74 (5.8)
Slovenia	116 (1.1)	30 (2.4)
Characteristics of transplanted patients		
n	6335	623
National MELD at delisting	22 (IQR: 16–29.4)	23 (IQR: 15–31)
Lab-MELD at delisting	16 (11–24)	16 (10–23)
Exception MELD points	2140 (33.7)	211 (33.2)
Standard exception	1989 (31.4)	172 (27.6)
Nonstandard exception	147 (2.3)	35 (5.6)
Split transplantation	122 (1.9)	45 (7.2)
DCD transplantation	525 (8.3)	46 (7.4)

Categorical variables are presented as absolute numbers and (percentage), and continuous variables are presented as median and (IQR). Lab-MELD based on actual values; national MELD may include exception points.

DCD, donation after circulatory death; HBV, hepatitis B virus; HCV, hepatitis C virus; IQR, interquartile range; MELD, Model for End-stage Liver Disease.

### Descriptive Analysis of Recipient Weight and Transplantation Probability

**FIGURE 1.**
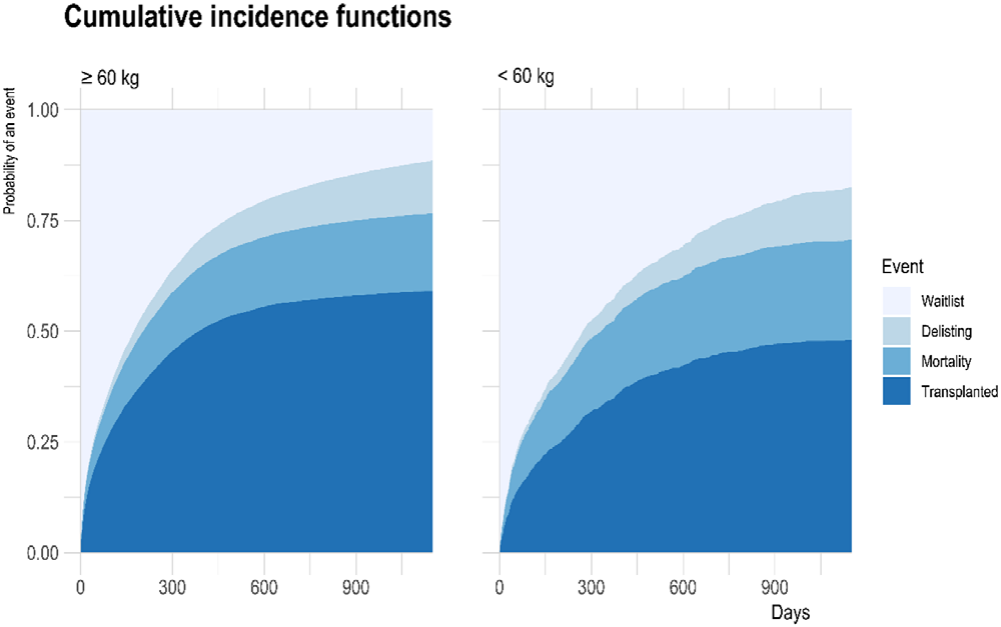
Stacked cumulative incidence plots for waitlisted patients. Cumulative incidences of transplantation, mortality, and delisting are shown for a period of 3 y as measured from listing onwards.

Stacked cumulative incidence plots for patients with a low bodyweight are presented in Figure 1. Patients with a low bodyweight (<60 kg) had a lower chance of liver transplantation. This resulted in increased waitlist mortality and delisting. At the end of follow-up, the percentage of transplanted, delisted, and deceased patients was 49.1%, 17.9%, and 24.3% for patients with a bodyweight <60 kg (n = 1267), respectively, versus 60.1%, 15.1%, and 18.6% for patients with a bodyweight ≥60 kg (n = 10 520). Reasons for delisting are summarized in Table 2. Median time spent on the waitlist was 175 d (IQR: 42–475.2) for patients with a bodyweight ≥60 kg versus 272 d (IQR: 65.5–731) for patients with a bodyweight below <60 kg.

**TABLE 2.** Reasons for delisting

n	Weight ≥60 kg	Weight <60 kg
Transplanted	10 520	1267
Deceased	6335 (60.2)	623 (49.2)
Delisted	1958 (18.6)	308 (24.3)
Reason for delisting		
Patient unfit for transplantation	512 (4.9)	55 (4.3)
Progression of malignant disease	214 (2.0)	15 (1.2)
Recovered liver disease	498 (4.7)	105 (8.3)
Other/unknown	349 (3.3)	50 (3.9)

Categorical variables are presented as absolute numbers and (percentage).

### Results of Cause-specific Time-dependent Cox Regression Analysis

**FIGURE 2.**
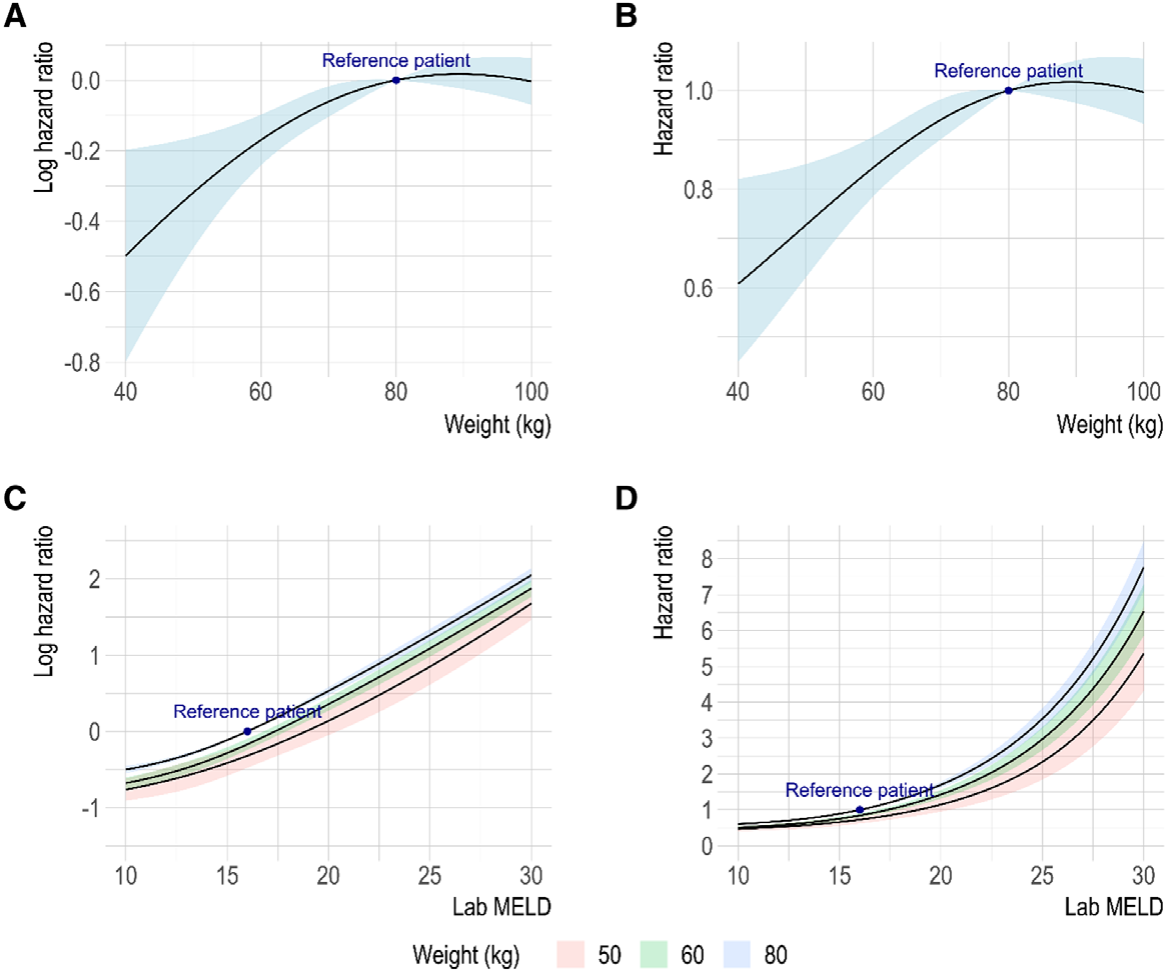
The effect of recipient bodyweight and lab-MELD score on transplantation chance. Colored bands represent 95% confidence intervals. The model included lab-MELD score (time-dependent) recipient, bodyweight, blood type, recipient age, and listing for malignant disease. Lab-MELD score and recipient bodyweight were fitted as interaction and with a natural spline with 3 degrees of freedom. Simulated effects were calculated relative to a reference patient with a bodyweight of 80 kg, blood type A, nonmalignant disease, and a lab-MELD score of 16. A, Effect of bodyweight on relative transplantation chance on the log hazard scale. B, Effect of bodyweight on relative transplantation chance as hazard ratio. C, Effect of lab-MELD on relative transplantation chance stratified by weight on the log hazard scale. D, Effect of lab-MELD on relative transplantation chance stratified by weight as hazard ratio. lab-MELD, lab-Model for End-stage Liver Disease.

Results of the regression analysis are presented graphically to adequately represent nonlinear effects. Lab-MELD scores were fitted as time-dependent variable with the inclusion of all available updated scores. Lab-MELD score was fitted as interaction with bodyweight. The model concordance was 0.736 and did not further improve by including natural splines with more degrees of freedom; model parameters are presented in **Table S1** (**SDC**, http://links.lww.com/TP/C886). Increasing lab-MELD score was associated with an increasing chance of transplantation (Figure 2C and D). To explore the effect of bodyweight on transplantation chances, the interaction between lab-MELD score and bodyweight was explored (Figure 2A and B). For patients up to approximately 70 kg, a higher bodyweight was associated with a higher chance for transplantation. For patients with a bodyweight of 70 kg and higher, chances for transplantation remained reasonably stable. Increasing lab-MELD score was associated with increasing chances for transplantation regardless of bodyweight, but for patients with low bodyweights, chances remained lower compared with their average-weight counterparts (Figure 2). Patients with a bodyweight of 60 kg or 50 kg needed to attain a higher lab-MELD score to attain similar chances for transplantation. For low-weight patients, the additional lab-MELD points required to attain similar chances compared with 80 kg individuals decreased as the total lab-MELD score increased (Table 3). Depending on the MELD score, 60-kg and 50-kg transplant candidates required, respectively, 1.1 to 2.8 and 2.1 to 4.0 additional lab-MELD points compared with >80-kg candidates with similar chances for transplantation. Expressed in a hazard ratio, chances for transplantation for 50-kg, 55-kg, and 60-kg patients were approximately 0.84 (95% CI, 0.77-0.91), 0.76 (95% CI, 0.67-0.87), and 0.68 (95% CI, 0.56-0.82) as compared with 80-kg reference patients with a lab-MELD score of 20 (Table 4).

**TABLE 3.** Estimated lab-MELD scores for patients with equal chances for transplantation, stratified by weight

Relative chance for transplantation (log hazard)	80 kg (reference)		60 kg			50 kg	
	MELD score	MELD score		MELD score difference	MELD score		MELD score difference
−0.501	10	12.8		2.8	14		4.0
−0.113	15	16.5		1.5	17.9		2.9
0.530	20	21.2		1.2	22.9		2.9
1.266	25	26.1		1.1	27.5		2.5
2.049	30	31.1		1.1	32.1		2.1

Lab-MELD scores for patients with equal chances of transplantation of different bodyweight were calculated based on the Cox model. For the simulation, a reference patient with a bodyweight of 80 kg, blood type A, 56 y of age with nonmalignant disease, and with a lab-MELD score of 16 was used.

MELD, model for end-stage liver disease.

**TABLE 4.** Relative chance for transplantation at different lab-MELD scores stratified by weight

Lab-MELD	80 kg	60 kg	55 kg	50 kg
		HR (95%CI)	HR (95%CI)	HR (95%CI)
10	Reference	0.84 (0.78–0.90)	0.80 (0.73–0.89)	0.77 (0.66–0.89)
15	Reference	0.84 (0.78–0.91)	0.79 (0.70–0.89)	0.74 (0.63–0.88)
20	Reference	0.84 (0.77–0.91)	0.76 (0.67–0.87)	0.68 (0.56–0.82)
25	Reference	0.84 (0.76–0.94)	0.75 (0.63–0.89)	0.66 (0.51–0.86)
30	Reference	0.84 (0.76–0.93)	0.77 (0.66–0.90)	0.69 (0.54–0.88)

HRs representing the chance for transplantation relative to an 80-kg patient. For the simulation, a reference patient with a bodyweight of 80 kg, blood type A, and age of 56 y was used.

HRs, hazard ratio; MELD, model for end-stage liver disease.

### Donor–recipient Size Matching

The mean donor–recipient bodyweight ratio was 0.98 (SD 0.21) for candidates with a bodyweight ≥60 kg and 1.27 (SD 0.32) for patients with a bodyweight <60 kg (Table 5). Patients with a low bodyweight were transplanted outside the 20% weight matching criteria in 59% of cases, and patients with a bodyweight ≥60 kg were transplanted outside these criteria in only 8% of cases.

**TABLE 5.** Donor and recipient bodyweight matching for transplanted patients

	Weight ≥60 kg	Weight <60 kg
n	6335	623
Donor weight	80 (70–90)	65 (60–75)
Recipient weight	80 (71–92)	54 (51–57)
Donor–recipient bodyweight ratio	0.97 (0.84–1.11)	1.25 (1.11–1.41)
Transplanted outside weight matching criteria (±20%)	892 (14.1)	370 (59.4)

Categorical variables are presented as absolute numbers and (percentage), and continuous variables are presented as mean and (SD).

### Characteristics of Transplanted Patients

**FIGURE 3.**
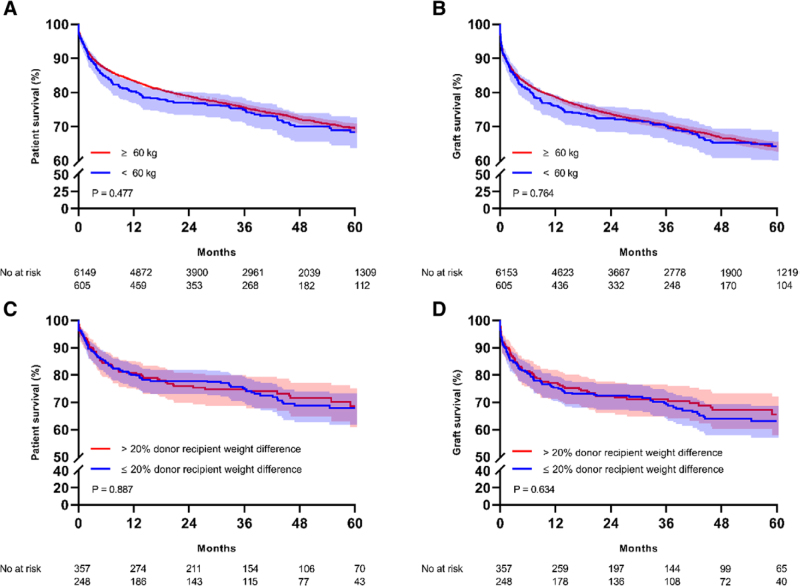
Kaplan–Meier curves of posttransplant graft and patient survival. Kaplan–Meier curves of posttransplant patient and graft survival. Colored bands represent 95% confidence intervals. A, Posttransplant patient survival is presented for recipients with a bodyweight ≥60 kg and <60 kg. B, Posttransplant graft survival is presented for recipients with a bodyweight ≥60 kg and <60 kg. C, Posttransplant patient survival for recipients <60 kg who received a graft from a donor with >20% weight difference vs ≤20% weight difference are presented. D, Posttransplant graft survival for recipients <60 kg who received a graft from a donor with >20% weight difference versus ≤20% weight difference are presented.

Characteristics of transplanted patients and post-transplant patient survival are presented in Table 1 and Figure 3. Median national MELD score at delisting was 22 (IQR 16–29) for patients with a bodyweight ≥60 kg versus 23 (IQR 15–31) for patients with a bodyweight <60 kg. In patients with a low bodyweight, nonstandard exceptions occurred more frequently (5.6%) compared with patients weighing >60 kg (2.3%). Additionally, split liver transplantation was performed 3.7 times as often in patients weighing <60 kg. Post-transplant patient survival was equal for low-weight and average-weight patients (Figure 3). Low-weight patients who were matched to relatively larger donors (>20% weight difference) showed equal posttransplant patient survival compared with patients matched to similar-sized donors.

## DISCUSSION

This study is the first to assess waitlist dynamics of small adult patients throughout the Eurotransplant region. The current analysis was corrected for actual lab-MELD update scores to correct for prioritization on the waitlist. Considering current data, small adults with a low bodyweight are significantly disadvantaged for timely receipt of a size-matched liver graft. This relative disadvantage increased with decreasing bodyweight and increasing MELD scores. This further implies that low-weight patients require a higher lab-MELD score, and therefore further progression of liver disease, to attain equal chances of transplantation compared with average-weight candidates. The aforementioned effects resulted in longer median waiting time, increased waitlist mortality rates, and increased delisting rates for small adult patients.

Recipient size is infrequently explored as a factor of potential importance in organ allocation. Nevertheless, the negative effect of small recipient size on allocation chances is well known in the transplant community and has been quantified before outside the Eurotransplant region.^1-4^ The degree to which smaller recipients are disadvantaged may differ as a result of differences in the demographic distribution of donor and recipient size within a transplant program. For example, similar to the current report, disadvantages have been reported in the United Kingdom, whereas this effect was less profound in the United States where the number of small patients listed on the liver transplantation waitlist is lower as compared with the number of small donors.^1^ In the current study, the lab-MELD update scores were used to adequately quantify the disadvantage corrected for prioritization on the waitlist. It appears that when corrected for prioritization on the waitlist, the disadvantage for small patients remains significant. The disadvantage of small adults may be explained by several factors. Within the Eurotransplant region, the number of small transplant candidates is smaller compared with the number of small donors, approximately 10.7% of transplant candidates had a bodyweight below 60 kg, whereas among the donor pool, only 6.9% of available donors had a bodyweight of 60 kg or lower. Additionally, small adults may compete with pediatric liver transplant candidates who are prioritized on the waiting list. Furthermore, average-weight individuals may be size matched to donors of both larger and smaller size, whereas small candidates will predominantly receive larger liver grafts, due to the weight distribution of available donors. Therefore, the eligible donor pool for average-sized patients is larger compared with small patients. The three latter effects will result in fewer available liver grafts suitable for small adult patients.

Considering the average weight of male and female patients listed for liver transplantation, the disadvantage related to small recipient size will predominantly affect female candidates and could therefore be considered one of the important reasons explaining previously observed gender inequalities in the allocation of liver grafts.^2,6-8,10^ Another explanation for the disadvantage of female transplant candidates may be the lower average serum creatinine in females, which causes for an underestimation of the MELD score.^16,17^ Prior publications have explored a correction for this inherent difference between male and female candidates.^16,17^ In the present study, we corrected for lab-MELD score without additional correction for creatinine, as this is not routinely applied in the Eurotranplant region. Moreover, we showed a disadvantage related to recipient size increasing with higher lab-MELD scores.

Considering observed donor size-matching for small adult patients, a number of important observations become apparent. Low-weight candidates are matched far more liberally compared with their average-weight counterparts. In fact, nearly 60% of candidates <60 kg received a donor graft from larger donors with >20% weight difference. This resulted in a relatively high average donor–recipient bodyweight ratio of 1.27. Graft weight was unfortunately not available in our database; therefore, graft-versus-recipient weight ratios could not be provided. Accepting relatively larger liver grafts as compared with the recipient size may have negative consequences due to technical concerns. The subdiaphragmatic space may be too small to facilitate enough space for implanting the larger graft and may result in mechanic obstruction potentially compromising venous outflow.^9-11^ However, when fulfilling specific matching criteria, use of a larger graft can be performed safely.^10^ Our data support the latter, as patient survival in low-weight patients who received a liver graft from a relatively larger donor was similar to those recipients who received a liver graft from a size-matched donor. Whether or not technical difficulties were encountered and their possible repercussion for morbidity could unfortunately not be assessed using ET registry data.

Potential solutions to address the disadvantages that small adults face on the waiting list include changes to the allocation system, the use of split or living donation or more liberal donor–recipient bodyweight matching. One may argue MELD exception points may equalize chances for this patient group. However, many other recipient factors influence chances for transplantation such as blood type and indication for liver transplantation. The use of exception points to overcome inherent inequalities may be complex and inconsistent with an allocation system based primarily on recipient urgency. Another solution could be to increasingly use split liver transplantation and living- donor liver transplantation (LDLT).^13,14^ In contrast to whole liver transplantation, LDLT and split liver transplantation are usually more accessible to small recipients, who may have a lower risk for small-for-size syndrome. Therefore, it might be beneficial to list small adult patients in transplant centers with an LDLT program or combined pediatric and adult transplantation program.^1^ Another less frequently applied technique is full-left full-right split liver transplantation, which may facilitate split transplantation for 2 adult patients.^18-21^ In current cohort, split liver transplantation was indeed performed 3.7 times more often in small adult recipients.

Apart from the use of split or LDLT transplantation, it might be important to standardize and change size matching itself. Even though size matching was applied liberally for low-weight patients, these patients still appear disadvantaged on the waitlist. Therefore, it is important to realize that applying size-matching criteria too strictly may further impair access to transplantation for this group of patients. Different methods for size matching have been previously proposed. Size matching could be performed based on a formula estimating the standardized liver volume (sTLV = −794.41 + 1267.28 ·body surface area [m^2^]).^22^ A donor–recipient sTLV ratio below 1.25 has been reported to facilitate safe transplantation.^10^ Transplantation beyond the 1.25 sTLV threshold may be associated with an increased risk for early allograft dysfunction; however, this did not result in increased graft loss.^10^ An alternative approach would be to use a body surface area (BSA) estimate defined as follows: $BSA=\frac{\sqrt{weight\left(kg\right)\times height\left(cm\right)}}{3600}$, this estimate would facilitate safe transplantation if the donor–recipient BSA ratio remains below 1.25.^11^ Higher ratios are associated with a slightly increased risk for graft loss.^11^ Although these alternative size-matching methods may ensure safe transplantation, it remains unclear if such methods would result in increased number of available donors for small recipients.

An alternative approach to size matching based on donor and patient size is to use actual size matching based on radiological evaluation. In a single-center experience, radiological evaluation to compare donor and recipient size was implemented in case of a high BSA mismatch.^23^ Radiological evaluation of the largest posterior-anterior distance of the liver, distance from the chest wall to the portal vein, and distance from the confluence of the portal vein to the suprahepatic caval vein were compared with donor and recipient. Based on these measurements, transplants could proceed if the space was considered sufficient; this resulted in safe transplantation in case of donor–recipient BSA matches >1.25. Considering the disadvantage of small adults and frequent use of larger liver grafts in these patients, radiological evaluation of donor and recipient to safely extent size-matching criteria warrants further exploration. Additionally, in theory, volumetric evaluation of the recipient subdiaphragmatic space and the donor liver may be used to provide additional information on size matching; however, to our knowledge, no prior study attempted this.

### Limitations

This study has several limitations. First of all, the survival status of delisted patients was not recorded; therefore, actual effects on survival from the moment of listing onwards, regardless of transplantation, could not be studied. Second, the analysis included all lab-MELD score updates; however, when transplantation was facilitated by exception points, these additional points were not captured within the time-dependent model. Although the percentage of exception MELD was higher in small versus normal-weight recipients, the absolute number was still low, and therefore, the total effect of these additional MELD points on the overall outcome is considered to be minimal. Third, for the descriptive analysis, 2 weight categories were used for illustrative purposes, and a cutoff of 60 kg was chosen, because based on the Cox model, a substantial disadvantage in chances for transplantation occurred when bodyweight dropped below 60 kg. However, from a bodyweight of 80 kg and lower, bodyweight was linearly associated with a lower chance for transplantation. Finally, the inclusion period ranging from 2010 until 2015 was chosen to ensure that the final outcome of most included patients was known. However, this period may not capture the effect of recent developments, such as machine perfusion, allowing for extension of the donor pool through increased usage of extended criteria grafts. Finally, the Eurotransplant region as a whole was studied; therefore, differences between countries within the Eurotransplant region or subtle differences in included centers were not captured.

## CONCLUSION

Small adult recipients with a bodyweight below 60 kg are disadvantaged on the waitlist to receive a timely size-matched whole liver graft within the Eurotransplant region. This disadvantage results in increased waiting times, delisting rates, and waitlist mortality. Strict recipient–donor size matching may further decrease access to transplantation for this patient population, and alternative methods to safely extent size-matching criteria such as radiological evaluation of donor and recipient should be explored.

## Supplementary Material
