# Supplementary material for: Quantifying the Disadvantage of Small Recipient Size on the Liver Transplantation Waitlist, a Longitudinal Analysis Within the Eurotransplant Region
Source: Transplantation. 2023 Nov 13;108(5):1149–56. doi: 10.1097/TP.0000000000004804 (PMC11042512; doi:10.1097/TP.0000000000004804)
Supplement: Supplementary file 1 [file tpa-108-1149-s001.pdf]

Table S1. parameters of cox model with time dependent Lab-MELD updates

Variable	Coefficient (log HR)	HR (exp coef)	95% CI	*P*
Lab-MELD (natural cubic spline; estimates do not have a direct clinical interpretation)	1.08	1.08	(0.14 - 8.33)	0.94
	24.88	24.88	(2.12 - 292.52)	**0.01**
	104.99	104.99	(25.57 - 431.12)	**<0.001**
Listing weight (First natural spline)	2.1	2.1	(1.24 - 3.57)	**0.01**
Listing weight (Second natural spline)	1.54	1.54	(0.12 - 19.47)	0.74
Listing weight (Third natural spline)	0.45	0.45	(0.06 - 3.35)	0.44
Recipient blood type A	(reference)			
Recipient blood type AB	2.21	2.21	(1.97 - 2.46)	**<0.001**
Recipient blood type B	1.00	1.00	(0.92 - 1.08)	0.91
Recipient blood type O	0.84	0.84	(0.79 - 0.89)	**<0.001**
Listing age	1.01	1.01	(1.01 - 1.01)	**<0.001**
Listing for malignant disease	2.04	2.04	(1.9 - 2.18)	**<0.001**
Interactions lab MELD and listing weight				
(natural cubic spline; estimates do not have a direct clinical interpretation)	1.16	1.16	(0.46 - 2.91)	0.75
	0.85	0.85	(0.25 - 2.88)	0.79
	0.89	0.89	(0.46 - 1.69)	0.71
	79.76	79.76	(0.69 - 9184.61)	0.07
	1.69	1.69	(0 - 612.66)	0.86
	0.62	0.62	(0.03 - 14.24)	0.76
	28.33	28.33	(1.18 - 680.18)	**0.04**
	1.61	1.61	(0.02 - 138.57)	0.84
	1.43	1.43	(0.28 - 7.28)	0.67
